# Supplementary material for: Microbial Diversity, Co-Occurrence Patterns, and Functional Genes of Bacteria in Aged Coking Contaminated Soils by Polycyclic Aromatic Hydrocarbons: Implications to Soil Health and Bioremediation
Source: Microorganisms. 2025 Apr 10;13(4):869. doi: 10.3390/microorganisms13040869 (PMC12029627; doi:10.3390/microorganisms13040869)
Supplement: Supplementary file 1 [file microorganisms-13-00869-s001.zip › microorganisms-3510540-supplementary.pdf]

Supplementary Materials

Table S1 PAHs contents in sample soil（mg/kg）

	rings	Coking production area				Office area			
		S1	S2	S3	S4	S5	S6	S7	S8
Nap	2	3.35	5.18	7.09	2.43	0.40	0.16	0.15	0.16
SD		0.63	1.07	3.02	0.15	0.11	0.05	0.04	0.06
Acy	3	6.65	10.27	20.13	2.22	0.88	0.58	0.08	0.34
SD		1.16	6.66	3.09	0.38	0.17	0.13	0.06	0.15
Ace	3	7.70	2.73	3.40	0.17	0.60	0.20	0.00	0.07
SD		1.28	1.69	0.43	0.12	0.29	0.00	0.00	0.05
Flu	3	8.70	10.96	10.79	0.59	0.73	0.26	0.04	0.17
SD		3.27	12.41	4.04	0.08	0.26	0.04	0.06	0.04
Phe	3	31.07	41.40	59.77	4.00	2.33	1.77	0.43	1.87
SD		16.24	36.08	14.24	0.80	0.50	0.12	0.17	0.87
Ant	3	9.00	16.23	27.37	1.63	0.90	0.73	0.07	0.43
SD		4.03	15.06	5.57	0.42	0.16	0.12	0.05	0.12
Flua	4	28.23	44.60	68.40	8.53	2.20	2.53	0.40	2.17
SD		15.35	28.10	8.62	2.41	0.14	0.21	0.28	0.68
Pyr	4	23.90	38.90	62.30	8.30	2.47	2.60	0.50	1.87
SD		12.91	24.89	6.07	2.35	0.21	0.45	0.14	0.39
BaA	4	11.90	22.50	45.97	4.87	1.67	1.63	0.23	0.97
SD		6.09	16.50	5.18	1.59	0.39	0.37	0.09	0.17
Chr	4	10.67	19.47	43.30	4.90	1.67	1.63	0.30	1.10
SD		5.34	13.63	4.86	1.39	0.33	0.29	0.08	0.37
BbF	5	19.03	34.93	75.40	10.00	2.73	2.67	0.47	1.50
SD		7.87	23.50	6.91	2.86	0.56	0.69	0.12	0.36
BkF	5	7.27	13.53	28.73	3.67	1.00	1.00	0.13	0.60
SD		2.94	9.12	3.01	1.06	0.22	0.24	0.09	0.14
BaP	5	13.30	25.23	51.13	6.60	1.73	1.77	0.27	1.00
SD		5.62	17.41	5.46	2.00	0.40	0.37	0.12	0.22
IcdP	6	6.20	11.20	24.30	3.87	0.67	0.77	0.10	0.60
SD		2.15	7.02	1.87	1.11	0.09	0.05	0.08	0.24
DahA	5	1.60	2.73	6.47	0.87	0.23	0.20	0.00	0.10
SD		2.15	7.02	1.87	1.11	0.09	0.05	0.08	0.24
BghiP	6	5.30	9.23	19.50	3.73	0.60	0.77	0.10	0.60
SD	-	1.64	5.38	1.24	1.03	0.08	0.17	0.08	0.24
∑PAHs	-	193.87	309.11	554.05	66.38	20.81	19.27	3.27	13.55
LMW	-	66.47	86.78	128.55	11.05	5.85	3.70	0.77	3.05
HMW	-	127.40	222.33	425.50	55.33	14.97	15.57	2.50	10.50
LMW/%	-	34.28	28.07	23.20	16.64	28.09	19.20	23.63	22.49

HMW/%	-	65.72	71.93	76.80	83.36	71.91	80.80	76.37	77.51

SD: Standard Deviation

LMW: low molecular weight

HMW: high molecular weight

Table S2 Mann-Whitney Test

N	8
Mann-Whitney U	.000
Wilcoxon W	10.000
Z	-2.309
Sig. (2-tailed)	0.021

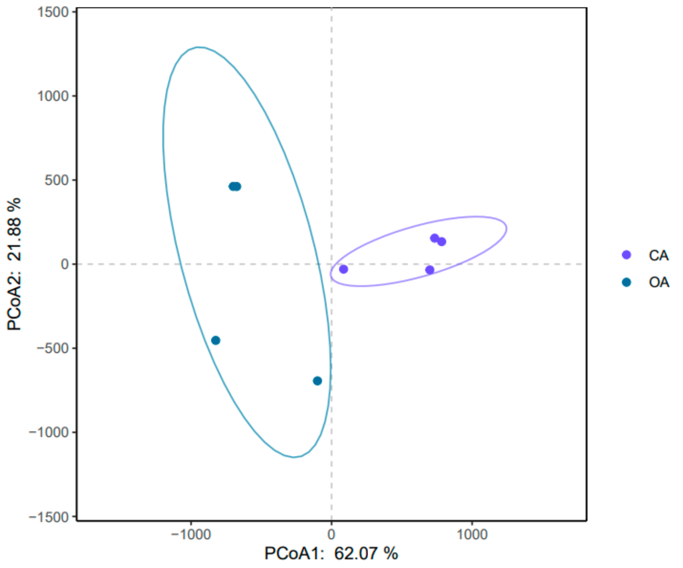

Figure S1 Principal coordinates analysis of Phylum

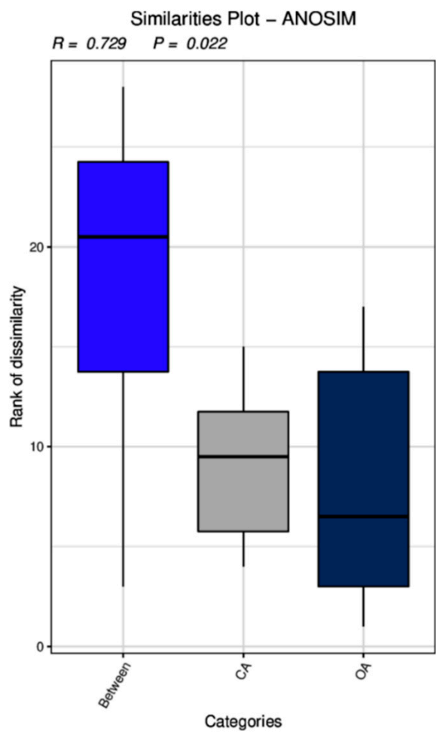

Figure S2 Anosim analysis based on species level

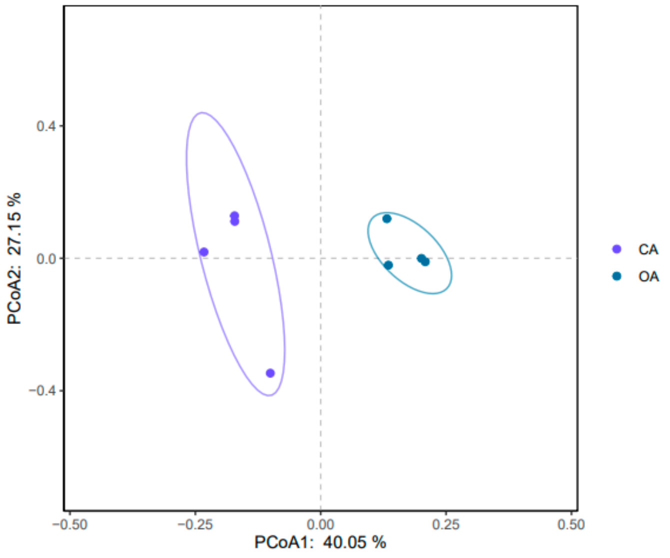

Figure S3 PCoA analysis of phosphorus functional genes

The extraction and sequencing methods of total DNA:

DNA libraries were generated. The reads can be found in the table below. The reads of all the samples were merged and assembled using MEGAHIT [1][2], a splicing software based on the principle of De-Brujin graph, to construct the De-Brujin graph based on the overlap relationship between kmer, to obtain the contigs, and to filter the contigs with more than 800 bp for counting and for subsequent analysis. Contigs above 800 bp were screened and used for subsequent analysis.

Sequencing data statistics table

Sample name	Total Reads	Clean Reads	Percentage	Clean bases	GC Content	%>Q20	%>Q30
S1_1	48,580,434	48,571,996	99.98%	7,282,641,610	65.09%	96.14%	89.03%
S1_1	47,778,620	47,772,988	99.99%	7,163,107,438	65.25%	95.97%	88.76%
S1_3	52,731,470	52,725,802	99.99%	7,906,344,016	65.53%	95.98%	88.82%
S2-1	40,727,424	40,722,936	99.99%	6,106,128,726	64.87%	95.71%	88.22%
S2-2	54,591,794	53,902,348	98.74%	8,083,004,552	64.55%	95.72%	88.4%
S2-3	50,850,352	50,843,836	99.99%	7,623,372,960	64.37%	96.91%	91.07%
S3-1	41,961,232	41,953,566	99.98%	6,291,461,070	65.47%	96.19%	89.22%
S3-2	42,736,634	42,728,874	99.98%	6,407,080,880	65.23%	95.98%	88.81%
S3-3	56,455,606	56,445,454	99.98%	8,464,690,396	65.32%	95.94%	88.6%
S4-1	45,177,324	45,170,482	99.98%	6,773,885,758	65.23%	95.86%	88.46%
S4-2	46,984,536	46,976,016	99.98%	7,044,568,390	65.5%	96.25%	89.46%
S4-3	49,363,998	49,356,502	99.98%	7,401,512,834	65.45%	95.89%	88.53%
S5-1	87,749,804	83,632,516	95.31%	12,540,402,550	64.54%	94.91%	87.38%
S5-2	100,085,012	95,275,222	95.19%	14,286,035,614	64.4%	95.68%	89.43%
S5-3	82,287,422	78,336,512	95.2%	11,746,019,794	64.46%	94.77%	86.94%
S6-1	70,926,706	67,635,298	95.36%	10,141,893,770	64.09%	94.56%	86.51%
S6-2	49,331,468	49,323,650	99.98%	7,395,720,058	63.98%	96.3%	89.65%
S6-3	48,561,618	48,553,638	99.98%	7,280,237,164	64.53%	96.64%	90.38%
S7-1	82,568,138	78,575,654	95.16%	11,782,022,888	63.89%	95.05%	87.61%

Sample name	Total Reads	Clean Reads	Percentage	Clean bases	GC Content	%>Q20	%>Q30
S7-2	47,210,322	47,203,400	99.99%	7,077,915,632	64.37%	96.08%	89.01%
S7-3	67,081,916	63,976,758	95.37%	9,592,969,164	63.53%	95.33%	88.72%
S8-1	67,508,106	64,360,108	95.34%	9,650,644,516	63.64%	94.79%	87.11%
S8-2	65,005,620	62,112,564	95.55%	9,313,322,622	63.33%	94.25%	85.9%
S8-3	76,437,506	72,842,512	95.3%	10,922,848,446	63.61%	95.14%	87.96%

Reference: [1].Li D, Liu C M, Luo R, et al. MEGAHIT: an ultra-fast single-node solution for large and complex metagenomics assembly via succinct de Bruijn graph[J]. Bioinformatics, 2015, 31(10): 1674-1676.
[2].Li D, Luo R, Liu C M, et al. MEGAHIT v1. 0: a fast and scalable metagenome assembler driven by advanced methodologies and community practices[J]. Methods, 2016, 102: 3-11.

The R language package for PCoA includes:

```
library('ggplot2')
library('reshape2')
library('dplyr')
library("optparse")
library(vegan)
library(ade4)
library(RColorBrewer)
library(vegan)
library(ggpubr)
library(ggrepel)
library(ggforce)
library(ggalt)
```

The R language package for Lefse includes:

```
library('ggplot2')
library('reshape2')
library('dplyr')
library(ade4)
library(vegan)
library("limma")
library(ggrepel)
library(ggforce)
library(ggplot2)
library("optparse")
```
